# VIRTUAL TELEHEALTH CLINICAL EXPERIENCE TO PREPARE STUDENT PHYSICAL THERAPISTS FOR PRACTICE IN A SNF

**DOI:** 10.1093/geroni/igac059.320

**Published:** 2022-12-20

**Authors:** Kathleen Manella, Kai Williams, Jon Anderson

**Affiliations:** Nova Southeastern University- Tampa Bay Regional Campus, Indian Rocks Beach, Florida, United States; Ensign Services, Inc, Cypress, Texas, United States; Ensign Services, Inc, San Antonio, Texas, United States

## Abstract

Student training was suspended in most post-acute settings in the United States due to the COVID-19 pandemic. Through a collaborative partnership between a university and a healthcare organization we offered a virtual telehealth clinical experience to prepare student physical therapists for practice in a skilled nursing facility (SNF) setting. 35 students engaged virtually for 80 hours with two clinical instructors. The curriculum included eight virtual learning modules along with four telehealth patient encounters that exposed students to interdisciplinary teams in the SNF context. Students completed a retrospective pre/post Self-Perception of Learning Survey. Results demonstrated students’ self-perception of learning was significantly increased implying an improved ability to practice in the SNF setting. This approach advanced student learning in patient care management, interprofessional practice, and telehealth practice in the SNF setting. Future work is planned to expand the program to include 20 physical therapy, occupational therapy, and speech and language pathology students.

